# Haemoptysis as a Presenting Manifestation of Hepatopulmonary Syndrome With Complete Resolution After Liver Transplantation

**DOI:** 10.1002/rcr2.70508

**Published:** 2026-02-10

**Authors:** Venkatkiran Kanchustambham

**Affiliations:** ^1^ Pulmonary & Critical Care Medicine Sanford Health and University of North Dakota Fargo North Dakota USA

**Keywords:** haemoptysis, hepatopulmonary syndrome, hypoxemia, intrapulmonary shunt, liver transplantation

## Abstract

Hepatopulmonary syndrome (HPS) is a pulmonary vascular complication of chronic liver disease characterised by intrapulmonary vascular dilatation and hypoxemia. Dyspnoea and orthodeoxia are classic features, while haemoptysis is rarely reported. We describe a patient with cirrhosis who presented with recurrent haemoptysis and progressive hypoxemia. Contrast‐enhanced transthoracic echocardiography demonstrated intrapulmonary right‐to‐left shunting, which resolved following orthotopic liver transplantation, with complete cessation of haemoptysis.

## Graphical abstract

Hepatopulmonary syndrome rarely presents with haemoptysis. This case demonstrates haemoptysis as a presenting manifestation of HPS, with intrapulmonary shunting documented on contrast echocardiography before liver transplantation and complete resolution after transplantation.

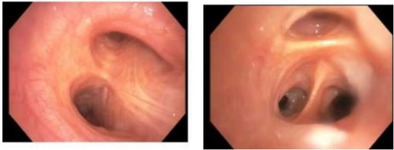

## 1 Introduction

Hepatopulmonary syndrome (HPS) is defined by the triad of chronic liver disease or portal hypertension, intrapulmonary vascular dilatation and impaired arterial oxygenation [1]. The prevalence among patients with cirrhosis ranges from 5% to 30% [1, 2]. Clinically, HPS typically presents with dyspnoea, platypnea–orthodeoxia, and progressive hypoxemia [1]. While uncommon, haemoptysis has been reported in isolated cases and remains an underrecognised manifestation [3, 4]. We report a case of haemoptysis as a presenting manifestation of HPS with complete resolution following liver transplantation.

## 2 Case Report

**FIGURE 1:**
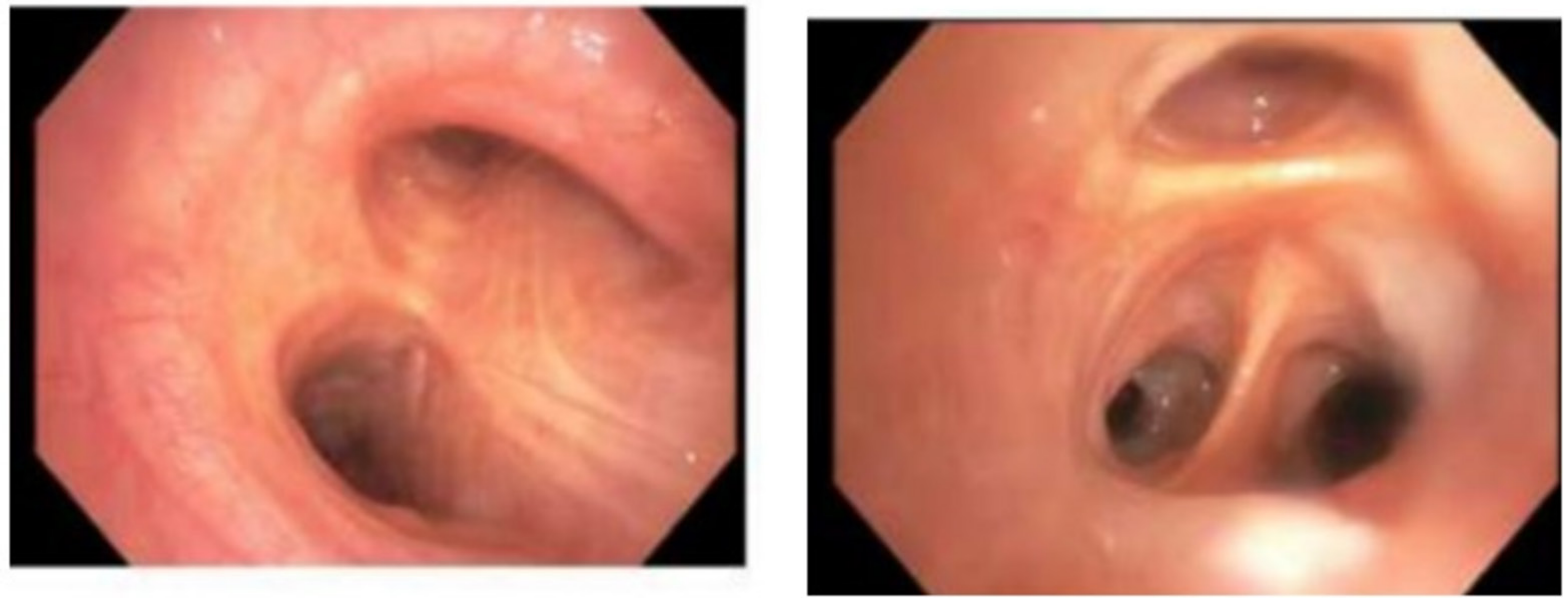
Bronchoscopy images demonstrating normal endobronchial anatomy without evidence of active bleeding or endobronchial lesion.

A patient with cirrhosis due to non‐alcoholic steatohepatitis presented with recurrent haemoptysis and progressive hypoxemia. Extensive evaluation including chest imaging, upper endoscopy, ENT examination and bronchoscopy failed to identify an alternative bleeding source. Flexible bronchoscopy demonstrated normal endobronchial anatomy without active bleeding or endobronchial lesions (Figure 1).

Arterial blood gas analysis confirmed severe hypoxemia with a widened alveolar–arterial gradient (Table 1). Transthoracic echocardiography with agitated saline contrast demonstrated delayed opacification of the left heart several cardiac cycles after right‐sided opacification, consistent with intrapulmonary right‐to‐left shunting (Video 1). The patient was diagnosed with hepatopulmonary syndrome and subsequently underwent orthotopic liver transplantation.

**TABLE 1:** Arterial blood gas prior to liver transplantation (room air).

Parameter	Value
pH	7.47
PaCO_2_ (mmHg)	25
PaO_2_ (mmHg)	34
Oxygen saturation (%)	86
Alveolar–arterial gradient	Markedly elevated
Oxygen source	Room air

**VIDEO 1:** Pre‐transplant transthoracic echocardiogram with agitated saline demonstrating delayed opacification of the left heart, consistent with intrapulmonary right‐to‐left shunting. Video content can be viewed at https://onlinelibrary.wiley.com/doi/10.1002/rcr2.70508.

Additional anatomic localisation of the shunt (e.g., pulmonary angiography) was not pursued because intrapulmonary shunting in hepatopulmonary syndrome is typically diffuse, and further localisation would not have altered management. At the time of diagnosis, the patient already met criteria for liver transplantation based on advanced liver disease with severe hepatopulmonary syndrome–related hypoxemia, with haemoptysis considered a secondary manifestation rather than the primary indication for transplantation.

Following transplantation, oxygenation progressively normalised and haemoptysis did not recur. Repeat contrast‐enhanced echocardiography demonstrated complete resolution of intrapulmonary shunting with absence of delayed left‐heart opacification (Video 2).

**VIDEO 2:** Post‐transplant transthoracic echocardiogram with agitated saline demonstrating absence of delayed left‐heart opacification, consistent with resolution of intrapulmonary shunting. Video content can be viewed at https://onlinelibrary.wiley.com/doi/10.1002/rcr2.70508.

## 3 Discussion

Hepatopulmonary syndrome (HPS) is defined by chronic liver disease or portal hypertension, intrapulmonary vascular dilatation, and impaired arterial oxygenation. Typical manifestations include dyspnoea, platypnea–orthodeoxia, and hypoxemia, while haemoptysis remains exceptionally rare [1].

A focused review identifies approximately six reported cases associating haemoptysis with HPS or related pulmonary vascular phenotypes. Schraufnagel et al. described haemoptysis with respiratory failure in severe HPS [3]. Gupta et al. reported haemoptysis resolving after liver transplantation [4]. More recently, Condon and Kanchustambham reported haemoptysis as a presenting manifestation of HPS with post‐transplant resolution [5]. Pasha et al. described haemoptysis in Abernethy malformation with secondary HPS, suggesting accelerated pulmonary vascular remodelling [6]. Lawton and Holmes‐Liew reported HPS in the setting of common variable immunodeficiency [7]. Sarac et al. described pulmonary arteriovenous fistula with haemoptysis, illustrating vascular mimics that may overlap with HPS physiology [8]. Shah et al. reported combined hepatopulmonary and portopulmonary vascular disease after paediatric liver transplantation [9]. Collectively, these remain isolated reports, with no cohort studies identifying haemoptysis as a recognised manifestation of HPS.

The pathophysiology of HPS involves pulmonary endothelial dysfunction with increased nitric oxide production and dysregulated angiogenic signalling, including vascular endothelial growth factor (VEGF), resulting in diffuse capillary dilatation, intrapulmonary arteriovenous communications and right‐to‐left shunting [1, 2]. Progressive vascular remodelling may create fragile microvascular networks susceptible to rupture under increased pulmonary blood flow and shear stress, providing a plausible mechanism for haemoptysis.

Previously reported cases often describe haemoptysis occurring late in the disease or in association with complex vascular or congenital abnormalities [3, 6, 8]. In contrast, the present case demonstrates haemoptysis as a presenting manifestation of HPS, with objective confirmation of intrapulmonary shunting and complete resolution of both hypoxemia and haemoptysis following liver transplantation, strengthening causal inference.

In this case, intrapulmonary shunting was confirmed by contrast‐enhanced transthoracic echocardiography. An additional anatomic localisation was not pursued because HPS‐related shunting is typically diffuse and would not have altered management once transplant candidacy was established.

Clinically, HPS should be considered in patients with chronic liver disease or portal hypertension who present with unexplained hypoxemia, orthodeoxia or otherwise unexplained haemoptysis after exclusion of common pulmonary, airway and gastrointestinal causes, as liver transplantation remains the only definitive therapy [10].

In conclusion, this case highlights haemoptysis as a rare but clinically significant presentation of hepatopulmonary syndrome. In patients with chronic liver disease, unexplained haemoptysis and hypoxemia should prompt evaluation for intrapulmonary shunting. Early recognition is essential, as liver transplantation can result in complete resolution of pulmonary manifestations.

## Author Contributions

Venkatkiran Kanchustambham was responsible for clinical management, imaging review and manuscript preparation.

## Funding

The author has nothing to report.

## Consent

The author declares that written informed consent was obtained for the publication of this manuscript, images and echocardiographic videos using the consent form provided by Respirology Case Reports.

## Conflicts of Interest

The author declares no conflicts of interest.

## Data Availability

The data that support the findings of this study are available from the corresponding author upon reasonable request.
